# Report on Small-Pox in Calcutta, and Vaccination in Bengal

**Published:** 1845-10

**Authors:** 


I. Report on Small Pox in Calcutta, (1833-4, 1837-8, 1843-44) and Vaccination in Bengal from 1827 to 1844. By *Duncan Stewart,* M.D. Surgeon, E.I.C. Superintendant-General of Vaccination. 8vo. pp. 280. Calcutta, 1844.

II. Observations on Variola Vaccina, or Cow-pock. By Sir *Matthew Tierney,* Bart. K.C.H. M.D. Brighton, 1845.

Well nigh half-a-century has elapsed since Jenner, by his *experimentum crucis* of unsuccessfully inoculating James Phipps after vaccination, offered to the world a demonstration of the greatest blessing it has ever yet received at the hands of medicine; for, however great difference of opinion may now exist as to the precise extent to which protection from a loathsome and fatal disease is secured, all will agree that the benefit derived from the practice is inestimable. Consider for a moment the position of mankind as regards Small-pox at the period of the discovery of Vaccination. The practice of Inoculation, after long struggling against popular apathy and clerical and medical opposition, had during the latter half of the 18th century become firmly established—the difference in the rate of mortality in the natural and communicated disease having attracted universal attention. For, while in the natural disease at least one in six died, and a large proportion of those who escaped were dreadfully disfigured and otherwise injured, in the inoculated the mortality was but 1 in 50, and, after the improvements introduced by the Suttons, 1 in 200 only, while the subsequent attack, of those inoculated, by the natural disease was a very rare event indeed. Yet, what was salvation to the individual became destruction to the community, so that at the period of the discovery of vaccination, by reason of the infinite multiplication of *foci* of infection, small-pox continued as rife as ever!—40,000 persons dying annually of the disease in England alone, with a population of nine million. Lettsom estimated the deaths in Europe from this cause at 210,000, and Bernoulli those of the world at large at 600,000 annually. Indeed the proportion of small-pox to other causes of death continued augmenting, for while it was but 72 per 1000 for half-a-century prior to the discovery of inoculation, it reached 85 per 1000 in the subsequent forty years, and mounted up, according to Sir Gilbert Blane, to 95 per 1000 during the last 30 years of last century.

Unfortunately, when we wish to exhibit to the best advantage the vast benefits which have been derived from vaccination, it is not to our own country we must have recourse for the necessary data; for, it is a fact, as strange as discreditable, that the birth-place of this brilliant discovery has been of all European countries the most negligent in availing itself of its blessings; and has never, until at least the last two or three years, given its preventive efficacy even the semblance of a fair trial. All has been left to the fitful operation of popular enthusiasm, with its alternations of action and apathy. The Report of the Registrar-General, reviewed in our last Number, exhibits some of the effects of this *laissez aller* system; for while deaths from small-pox amounted in France in 1842 to about 91

in 1,000,000; and those in Austria to 4619, 5189, and 4411 in 1840-1-2 in a population of above 21 million, in England they amounted, prior to the introduction of the present Vaccination Act, to from 9000 to 16,000 in a population of less than 13 million. The contrast might be rendered still greater were the comparison carried into those continental states, as Denmark, Sweden, &c. in which vaccination has been rendered peremptory. We have no statistical data for exhibiting the numbers of our infant population which yet remain unvaccinated; and it is only upon the visitation of an epidemic that our culpable negligence becomes fully revealed. It is true that the mere passing through our crowded streets, and observing the great rarity with which persons disfigured with the small-pox are met with, compared with heretofore, affords a rough but satisfactory proof of how much has already been accomplished; but the almost entire immunity which our naval and military services enjoy from a scourge which formerly ravaged them so fearfully, exhibits how much more yet may be done by adopting additional precautions, the necessity for which the returns of all the local registrars testify to, by their numerous entries of deaths of unvaccinated children.

No one, however, is now sanguine enough to believe that small-pox, to whatever extent it may be kept under, and its severity mitigated, by diligent and *universal* vaccination, will ever cease to be one of the occasional afflictions of the human race. But this affords us not the shadow of an excuse for neglect and remissness in the application to the greatest possible extent of the only means which approaches to the character of a preventive. The history of Vaccination in Sweden, in this point of view, is interesting. Dr. Retzius states* that, during the two first years of the present century, the kingdom of Sweden lost 18,089 inhabitants by the small-pox, and 6057 were annually swept away upon an average during the 18th century; but after the introduction of vaccination, in 1803, this mortality gradually diminished; so that while during the preceding half-century 15 per cent of the children were carried off by small-pox, and of every 11 deaths 1 occurred from this disease, in 1804-5, 1 child only out of 44 born, died of the disease, and in every 40 deaths 1 only was caused by small-pox. By 1810, in many portions of the country, full one-half the population were vaccinated, but small-pox prevailed in force every now and then, being especially propagated by mendicants and gypsies, and unqualified vaccinators. In 1813 the disease had made great head, and the popular opinion received a great shock in consequence of several of the vaccinated acquiring it. In 1816 a royal ordinance imposed a fine upon all persons neglecting to have their children vaccinated within the two first years of their lives, and restricted the performance of the operation to qualified persons. All persons, too, who had neither had the small-pox, or had been inoculated, were compelled under fine to be vaccinated within a certain period. The mortality from small-pox for the years 1819-23 only amounted to 0·0017 of the entire mortality. At the end of 1823, however, the small-pox, imported into the ports by some sailors, spread fearfully in the interior and affected fatally numbers who had been vaccinated,

* Gazette Medicale, 1843, No. 63.

inoculated, or had had the disease naturally. Of about 85,000 persons attacked, there died in 1824, 0·73 per cent.; in 1825, 1·34; in 1826, 6·735, and 1827, 0·7 per cent. But even this mortality is nothing in comparison with that resulting from the old epidemics; when 42 out of every 1000 inhabitants annually died of the disease, and 14 per cent. of the cases were fatal. During the years 1824-37 there died but 243 per annum of small-pox. In 1838 another epidemic, which also so severely preyed on other parts of Europe, again appeared, and in 1838-40 there were 54,870 persons attacked, of whom 4,389, *i. e.* 7·91 per cent. died—the disease affecting both those who had been vaccinated, and those who had had the disease naturally in childhood. From this period there have not been more than 250 cases per annum. Diligent Vaccination then would seem to have the power of maintaining a low average mortality, but not of completely protecting against occasional epidemics.

The diminution of mortality from Small-pox which occurred in *London* and its suburbs a year or two after the adoption of the provisions of the Vaccination Act in 1840, has been, by a visitation of the epidemic of last year, replaced by an augmentation. The numbers for the years 1838-44 were as follow:

| | 1838 | 1839 | 1840 | 1841 | 1842 | 1843 | 1844 |
|---|---|---|---|---|---|---|---|
| Small-pox . . | 3817 | 634 | 1235 | 1053 | 360 | 438 | 1804 |
| Measles . . | 588 | 2036 | 1132 | 973 | 1293 | 1442 | 1182 |
| Scarlatina . . | 1524 | 2499 | 1954 | 663 | 1224 | 1867 | 3029 |
| Pertussis . . | 2083 | 1161 | 1069 | 2278 | 1603 | 1908 | 1292 |

The deaths from Small-pox during the two first quarters of 1845 amounted to 727. We have placed in the above table the number of deaths caused by the three other most destructive diseases of children, from which it appears that Small-pox stands even yet third on the list; the totals of the seven years being as follow:—Scarlatina, 12,760; Pertussis, 11,394; Small-pox, 9,341; Measles, 8,646. The synopsis is interesting too in reference to the alleged greater predominance of these other forms of disease in those years when Small-pox carries off fewest victims.

Although many of the generalizations of the sagacious Jenner have been confirmed by subsequent experience and observation, the most important one, that vaccination is as perfect a protection against small-pox as small-pox itself, unfortunately must now be abandoned; for, although Dr. Baron, and a few other eminent practitioners in this country and in France, still maintain this opinion, an extensive examination of facts will certainly not confirm it. The authority of Jenner and the immunity of the early vaccinated, long indisposed professional men to the admission of cases of small-pox after vaccination, upon other grounds than idiosyncrasy or imperfect vaccination; although Drs. Copland and Brown, from contemplating several of these, predicted at an early period, that the protective power of vaccination would not prove permanent. Even now, it is the growing sense of insecurity which occupies the public mind that has forced the matter upon professional attention, and has extorted the acknowledgment of the necessity of stating the extent of immunity to be expected from vaccination with far greater circumspection than heretofore. The natural anxiety of the educated portion of the community has been much excited of late upon the subject, and the statistics of all recent epidemics show with what justice. Thus in thirty of these occurring in France, between 1816 and 1841, 5963 of the subjects of small-pox had been vaccinated. In an epidemic at Marseilles, 478 in 864 cases are said to have undergone vaccination. Of the 647 patients admitted into the London Small-pox Hospital in 1844, 312 had been vaccinated and presented genuine cicatrices, and 22 were said also to have been vaccinated, but did not present proper cicatrices.

Forced to admit that the original hopes excited by vaccination were too sanguine, their disappointment has been explained by observers in different modes. Thus, it has been said the vaccine virus may have degenerated in the course of its long-protracted employment. But this is a figment adopted by few, for the physical appearances and course of the disease engendered by it are precisely the same as in the time of Jenner; and it is those persons who were long ago vaccinated with virus, which according to this hypothesis should be possessed of greater efficacy, who are the subjects of small-pox, while those who have been operated upon recently are exempt. Then, again, we think far too much stress has been laid upon the faulty mode of conducting the operation as an explanation of the facility with which small-pox is subsequently contracted. In the immense majority of cases, where the vaccination succeeds at all, it pursues its course with regularity and is followed by the characteristic cicatrix; and there is not a semblance of proof that patients in whom some trifling difference in the local appearances, especially as regards the dates of the various changes, have occurred, are those whom small-pox especially affects. It is quite consistent with all analogy of other diseases that such differences should be much more striking; and we think the Provincial Medical Association, in their report on vaccination, have laid far too much stress upon these circumstances. Let it be remembered, too, that patients vaccinated by Jenner himself, and under his superintendence, have yet contracted small-pox.

If we are to believe the statements of the officers of Vaccine Institutions, the operation requires a dexterity, and the superintending its effects a sagacity, far beyond the competency of the surgeon qualified for the ordinary emergencies of his profession. We are no advocates for intrusting the diffusion of vaccination to ignorant and incompetent persons; but we must guard against this tendency always shewn by persons engaged in special occupations to exaggerate their difficulty and importance.

In the Report of the "Royal Jennerian and London Vaccine Institution," for 1845, we find the following elegant *morceau* from the pen, we presume, of its "Medical Director," Dr. Epps. After stating that they had vaccinated 6717 during the last year—truly a drop in the ocean in a metropolis with its two million souls—he proceeds—

"This increase establishes the high and increasing estimation, in which this Institution is held by the public; an estimation founded primarily upon the success attendant upon the vaccination performed at the Stations of the Institution; founded in part, it is to be feared, on the imperfection of the vaccinations performed by many surgeons, who practise vaccination, and consequent upon this, the spread of small-pox. Your Board of Managers have no wish to represent as incapable the majority of practitioners who vaccinate; but, having declared formerly their opinion that the Vaccination Act would be likely, by inducing surgeons unacquainted with vaccination to vaccinate, and thence likely to vaccinate inefficiently, to create by their vaccination a spurious confidence on the part of the vaccinated, that they are safe because vaccinated, when in reality they have not been vaccinated at all, and having found that small-pox has dreadfully increased (?) since the Act has come into force, they feel bound to refer to this explanatory fact; to enter their protest against the presumed inefficacy of vaccination, and to throw the want of protection upon the proper source of such want, namely, the ignorance of many practitioners who vaccinate, of proper vaccination. * * * The Board of Managers feel less hesitation in dwelling on this cause of the spread of small-pox because they know that there are numerous surgeons who do vaccinate properly (a list of such as belong to the Institution is furnished accordingly), and further, that genuine vaccine is to be obtained at the Royal Jennerian and London Vaccine Institution, and therefore it is the fault of medical men, if spurious vaccination exists; or, if existing, is not recognised and declared as such."

Dr. Gregory, a far higher authority, and a respecter at least of the Queen's English, even observes:—

"These cases further impress me with the great importance of restricting the number of those to whom the parochial system of vaccination (enjoined by the Vaccination Extension Act) is entrusted. I speak after 22 years' experience in the practice of vaccination: and I know that the choice of effective lymph requires much tact and discrimination, and that, except at Vaccine Stations, where considerable numbers congregate, such choice cannot be had, nor such knowledge acquired."

The explanation now adopted by most observers, save such as still believe Jenner infallible in his predictions, is probably the true one; viz. that the duration of the protection by vaccination is limited, the constitution re-acquiring its susceptibility for the small-pox after the lapse of a certain period of time.

To remedy the admitted imperfections of vaccination some consider that a more careful attention to its process will prove sufficient, which, however, is an opinion rapidly losing ground. Others believe that, by again having recourse to the cow, we shall acquire a more efficient virus. This suggestion has brought to light the fact that the genuine disease is itself of rare occurrence in this animal, and liable to be confounded with spurious, non-protecting, or even, as occurred in Bengal, infecting eruptions. M. Bousquet states that no genuine case of pock in the cow was observed in France prior to 1837. However, new supplies have now been obtained by Mr. Estlin at Bristol, at Passy near Paris, and elsewhere; and the local effects produced by the new virus have, in some cases, been more marked, but in others no-wise greater than those produced by the old vaccine. M. Fiard, speaking of the experiments at Paris, states that the greater activity at first manifested disappeared after a few years employment of the new virus.

Some practitioners regret that medical practitioners should not have been excepted from the operation of the clause of the Vaccination Act which renders *Inoculation* for the small-pox penal. Dr. Gregory, reasoning from the facts brought before him at the Small-pox Hospital (the experience of which institution he has in the most praiseworthy and indefatigable manner from time to time brought under the notice of the profession), recommends a practice in the propriety of which we cannot concur. In the year 1844, 647 patients were admitted, being a greater number than any year, except 1838, has furnished since the introduction of vaccination. Of these 647 persons, 312 had satisfactory vaccine cicatrices. The proportion of cases of small-pox after vaccination has been steadily increasing in the hospital from 1809, when it was 1 in 36, to 1844, when it was 1 in 2. Although 100 of the 312 had the mild varioloid disease, there were 24 of the remaining cases which died :—giving a mortality of 8 per cent. while the mortality after inoculation is but $\frac{1}{5}$, *i. e.* 1 in 500. Dr. Gregory therefore considers that the best course that could be pursued would be to inoculate as they approached puberty those persons who had been vaccinated in infancy.

In reply to this we may observe—1. That the absolute increase of small-pox in the community at large cannot be judged of by the limited experience of the London Small-pox Hospital, unless we are put in possession of the comparative increase of the population of the districts whence it is supplied with patients. 2. That the very extension of vaccination itself explains why the proportion of admissions of small-pox after vaccination is upon the increase; and we hope the time may come when none other will present themselves for admission. The truly important question is not what proportion do these bear to the admissions for the natural disease, but to the number of persons who have been vaccinated—a question the solution of which is not at present possible. 3. That this mortality of the vaccinated, derived as it is from the observation of patients taken from the lowest classes of society, exposed to every adverse hygienic circumstance, is far too high as applied to small-pox after vaccination in general. We have no statistical returns elucidatory of this question ; and Dr. Gregory's statement, in his letter to the Registrar-General, "The mortality by small-pox, as it occurs among the vaccinated, is 10 per cent." is derived solely from data furnished by the Small-pox Hospital. According to the French Report, presently to be noticed, the mortality is slight. Thus, in 5963 cases of small-pox after vaccination, occurring in France in 1816—41, there were but 62 deaths ; and of 2000 occurring in the violent Marseilles epidemic of 1828, only 45 died. At the Hospital La Pitié however, during the epidemic of 1825, the mortality was far greater. Of 682 admissions there were 162 persons who had been vaccinated, 88 in whom the success of vaccination was doubtful, and 432 who had not been vaccinated. There died 25 of the vaccinated and 148 of the non-vaccinated. Among the non-vaccinated were 14 instances of secondary small-pox, two of which only died. 4. The plan of inoculation, however beneficial to the individuals operated upon, would prove a very destructive one to the community. Again, in spite of whatever care might be taken, should we find centres of infection multiplying on every side, and thus should become engaged in the not very wise proceeding of undoing with one hand what we were doing with the other.

The only unexceptionable procedure would be the general adoption of the practice of *Re-vaccination*. Every medical man must be aware that the question of the propriety or necessity of this practice has excited much attention of late among the better-educated portion of the public, although its investigation has been unaccountably neglected by the profession in this country. To us there seems everything in favour of its adoption, and no one valid objection to urge against it. It has been said indeed that such adoption would unsettle the public mind in its faith in vaccination. Nor need this be regretted. The most fatal condition of the public mind, that which is really to be dreaded, and from which so much evil has already sprang, is *apathy*. Let public attention be fairly aroused, the merits of vaccination will then undergo renewed discussion and examination, and its more general adoption, even with an understanding of its limited protective power, must be the result. It is to the continental states, especially Prussia and Wurtemburg, that we are indebted for carrying on the experiment of re-vaccination upon a large scale, the results of which have been frequently detailed in the pages of this Journal.* In the former country, of 216,289 re-vaccinations during 1833-7, there were 84,516 successful; and of 44,000 in the latter country, 20,000 succeeded. Frequently, too, cases which failed on a first trial succeeded on a subsequent one. The precise proportion of successful cases has varied from 31 to 45 or 46 per cent.—the period between the ages of 10 and 30 being found that most certain of success. Of course, no one infers that the success of re-vaccination implies a liability to small-pox in an equal number of cases. The operation, in fact, in the hands of Heim, proved successful also in 32 per cent. of persons who had already had the small-pox—a proportion infinitely greater than that in which small-pox occurs a second time. But, although we are unable to state the exact proportion of the vaccinated persons, in whom re-vaccination succeeded at the rate of 34 per cent., who would otherwise have acquired small-pox on exposure, yet experience has shewn this might have been considerable; whereas, among the many thousands who have undergone re-vaccination in Prussia and Wurtemburg, an example of the occurrence of small-pox has only here and there been observed. Moreover, in the case of an epidemic breaking out, it has been found, in various localities, that immediate re-vaccination has *arrested its course*—individuals in whom the operation proved successful and those in whom it failed equally resisting the disease.

The French *Academie des Sciences* proposed prizes in 1842 for the best memoirs upon the present state of the vaccination question. On account of the great number (35) and size of the essays, the decision of the Commission deputed to examine them has been delayed until the present year. The Report prepared by M. Serres is a highly interesting document, and observers so well known as MM. Bousquet and Fiard are found among the successful competitors. Our last Periscope contained the conclusions, which the Commission, composed of MM. Magendie, Breschet, Dumeril, Roux and Serres, agreed upon; but the importance of the document demands of us a somewhat farther notice here.

The 1st question is couched as follows, "*Is the preservative virtue of the vaccine virus absolute or only temporary? In this latter case determine by precise experiments and authentic facts the period during which vaccination preserves from variola.*" The following are the replies to the queries,

---

* See especially Vols. 30, 31, 34.

preceded by a few interesting observations upon the *varioloid,* by M. Serres.

" This mitigating power is confirmed by the experience of every physician, who since the researches of Dr. Thompson of Edinburgh, published 1818-20, have recognized in the varioloid of the vaccinated, natural variola, deprived most frequently, by the vaccine, of the grave characters which render it so dangerous. By reason, however, of the importance, and to a certain point the novelty, of this acknowledged property of vaccinia, your Commission has deemed it useful to support by its own experience a fact which the public is ignorant of, and one which it is so necessary for it to become acquainted with. It has deemed it important to disengage this property from the vague expressions by which it has been designated, as, *modified, mitigated, shorter, benign, variola, &c.* and replace them by less equivocal characters, which allow of the determination in what and how vaccination modifies natural small-pox in so advantageous a manner.

" If we except the intermitting fevers there is no disease which progresses with more regularity than natural small-pox. The four periods which constitute it—the fever of incubation, the eruption, the suppuration, and the desiccation—succeed each other with an order and regularity that nothing can derange, not even complications, not even intercurrent diseases which modify their nature. What these cannot do vaccination effects. Its result is to arrest the periods of the disease and to cut it short: so that when it does not retain its preservative power against the inroads of the disease, it yet preserves it in its various periods. Thus when the varioloid papulæ appear in the vaccinated they do not suppurate. This is the simplest case of varioloid. At other times, suppuration in part commences but is suddenly suspended. Again, the suppuration may follow its course and abortion of the desiccation occur. It is remarkable that the abortion of the pustules is constantly followed by the arrest of their corresponding symptoms. It is on this account that the variola of the vaccinated is so little severe. This arrest of the development of the pustules modifies much their characters. Some have distinguished 5 and others 11 species of varioloid, and these might be yet farther multiplied, but uselessly so; for it is only the periods which they traverse, which offer any interest in the terminations of the variola in the vaccinated.

" The comparison of results enables us to draw three conclusions. 1. That the preservative power of vaccination is absolute and general for the eight and nine first years after its performance, and even to the 10th or 12th year, according to the experience of re-vaccinators. 2. When this age is passed, and especially under the influence of epidemics, a part of the vaccinated, but a part only, are liable to contract variola. 3. The greater number of the vaccinated are probably protected for life."

The *Second Question* is, *" Has the Cow-pox a more certain or permanent preservative power than virus already employed during a greater or less number of successive vaccinations?* The concurrent testimony of numerous observers proves that the local effects of the new virus are more marked; but it by no means follows that the preservative power is proportionate to the intensity of these. The vaccinations made with the new virus are of too recent a date to admit of any fair comparison being made.

" We may terminate this part of the question by citing two facts which prove the energy of the virus at a period when it was supposed to be enfeebled by reason of the diminution of the intensity of its local phenomena. 1. Small-pox appearing in the College at Sorèze attacked 40 pupils, of whom two only had not been vaccinated. Dr. Millon re-vaccinated all the others to the number of 300, when the contagion became suddenly arrested. 2. Small-pox, which prevailed at Mantua in 1831, penetrated into the Foundling Hospital, and 12 were attacked. Dr. Solera re-vaccinated the other 200 children and the disease disappeared."

We may observe, however, that these facts, occurring only in children, are not quite so conclusive as at first sight they would appear to be.

*Third Question.—Supposing the preservative power of the vaccine becomes enfeebled by time, should we renew it, and by what means?* Observation has proved that, not only the original but the recently-obtained virus fails to produce after a time the intense local effects which it did at first: so that if these are to be maintained, it is necessary to provide means for its renewal. Three means have been proposed. 1. To inoculate the cow with the grease of the horse or with human variola. 2. To restore its native strength to the vaccine virus by returning it to the cow from man. 3. To retake the vaccine at its source. The inoculation of the cow with the matter of grease, although frequently unsuccessful, has at other times succeeded, and deserves farther trial. The same may be observed of the inoculation of this animal with human variola. (No notice is taken of Mr. Ceeley's successful experiments). The employment of virus obtained from vaccinating the cow, has been stated by some as more successful in the proportion of 1 to 3, while others represent it as having produced no improvement. Might not satisfactory results be produced by first vaccinating the cow from man and then transmitting it through many successive cows? However, the Reporter adds, the means which is of all others to be preferred is that recommended by Jenner, viz. seeking a fresh supply from the cow.

"It seems to this Commission, that it will prove very useful to endeavour to propagate the natural cow-pock. To this end it believes it necessary to suggest to observers who may find it anew not to content themselves, as has hitherto been the case, with transmitting it to man, but also to endeavour to transmit it to other cows, and to collect it for preservation and diffusion, so that the vaccine may be renewed as often as possible. It equally recommends observers to describe carefully those eruptions of the cow, which by their characters resemble cow-pox, to expose the differential marks, so that it may be easily recognized, and that certainty given to this point of comparative medicine which its importance deserves."

### *Fourth Question.*

"*Is it necessary to vaccinate the same person several times? and, if so, after how many years should we have recourse to these new vaccinations?* The examination of facts leads to the conclusion, that vaccination does not always preserve from small-pox; but that the enfeeblement of its local phenomena does not diminish in the same proportion its preservative power. Thus, in augmenting this local intensity by the renewal of the virus we may hope for the conservation of its properties but not for their increase. Those vaccinated by the renewed virus will remain as the others liable to attacks of variola. Such attacks bear relation then not to the quality of the virus but to the age of its insertion, so that man is almost absolutely preserved until adolescence. After that period certain of the vaccinated are liable to attacks of variola until 30 or 35 years of age, after which period their preservation is absolute and almost certain.

Neither the intensity of the local or general symptoms, the multiplicity of the punctures or cicatrices, nor the appearance of these latter, upon which, upon the theory of Gregory, such great hopes had been founded, have furnished any certain index for the foundation of a prognostic."

*Re-vaccination* is the only means we have of distinguishing those of the vaccinated who are definitively protected from those who are so but temporarily, in whom the preservative power is enfeebled. The immense success attending this practice in Germany, led to repeated discussions in the French Academy of Medicine, the members of which had not their confidence in vaccination destroyed by these assemblages of figures, especially until their true value could be enquired into. The very different results obtained in different countries are remarkable; for while at Petersburgh the successful re-vaccinations only amounted to 3 per cent., and in Paris to 10, or with the new virus 20 per cent., they reached to 50 per cent. in Prussia, and in some parts of Wurtemburg to even 70 per cent! So too, even in Wurtemburg, while some portions of the country furnished this 70, others furnished 29 only, and the army but 34. The reporter believes that a great number of these cases have been set down as successful re-vaccinations which would not be so considered in France.

The last subject to be noticed is the *degree of liability the re-vaccinated possess of contracting small-pox*. Jenner made an observation which should not be neglected by re-vaccinators—that, although cow-pox or vaccination protects against the small-pox, and variola against the cow-pox, yet cow-pox is not always enabled to afford protection *against itself:* so that, in some individuals, the visible phenomena of vaccination may be reproduced several times. Re-vaccination has therefore not the important signification stated to belong to it by some; it is not a *variolometer*, and thousands of vaccinated persons, after having been exposed to the contagion of small-pox without acquiring the disease, yet may be vaccinated a second time with success. Moreover, persons have been extensively re-vaccinated who have had the small-pox.

These facts raised at first much opposition to re-vaccination, until the experience of its *power in arresting epidemics* overcame the arguments of all objectors in the north of Europe. The Commission, observing that small-pox has been endemic for the last twenty years in Paris, and believing re-vaccination to be, after a first vaccination, the only efficacious means that remains, urges its adoption in France. All observers who have extensively resorted to its employment are unanimous in acknowledging its benefits and recommending its extension; and the same approval is bestowed upon it by the whole of the competitors for the prizes.

Dr. Stewart's interesting work is divided into three parts. One of these treats of recent Epidemics of Small-pox in Calcutta, the second of an Epizootic Disease prevailing among the cows in the same city, and the third upon the History, State, and Prospects of Vaccination in Bengal. We will briefly advert to each of these.

1. *Small-pox in Calcutta.*—Dr. Stewart observes, that although cutaneous diseases are those which most prevail and prove most troublesome among the Native population of India, they have attracted little of the attention of the authors of diseases of that country.

"The oversight has been doubtless owing in some degree to the partial intercourse which English Physicians have with the lower orders of the people. With few exceptions the professional duties and avocations of Medical Officers in India bring them into acquaintance only with the native sick in Regimental, or in Gaol Hospitals. In Calcutta, a few practitioners have access to the houses and families of some of the upper classes of the wealthy Hindoos; and a smaller number still, in charge of Dispensaries, have opportunities of becoming familiar with the prevalent diseases of the *poor.* But these charities afford even to the most zealous and assiduous lover of his profession a very partial idea of the acute diseases of the country; for, except in cases of accident or of cholera, no in-door patients are received into these institutions; and of the hundreds who apply for advice and medicine daily, *all* are too poor to afford the hire of conveyance thither often or regularly; besides, in dangerous illnesses, few of course could bear this. Hence also in a great measure it happens, that among many thousand patients who have applied for and received advice and medicine at the various Calcutta Dispensaries since 1st Jan. to 1st of July, *not one single case of small-pox* has been seen, while the streets have been crowded every day with funeral processions, and the smoke of funeral piles on the banks of the river has tainted the morning gale for months past."

Although small-pox itself is not seen in the patients attending at the Dispensaries, cases exhibiting all its horrible sequelæ present themselves in abundance. Of 280 patients presenting themselves daily at the Park-street Dispensary, 12 on an average, chiefly children, attend on account of these!

The last epidemic of 1843-4, first commenced in November, during which month the Hindoo ghat registers reported 14 deaths from this cause. These augmented in Dec. to 58, in Jan. to 157, in Feb. to 455, in March to 963: and several Europeans having also fallen victims to the disease, a general panic prevailed. Small local hospitals for the admission of patients were established in different parts of the town, additional vaccinators employed, and advertisements inserted in the newspapers offering rewards to those parents who presented children bearing perfect vaccine crusts.

"But the Hospitals remained nearly tenantless, and, after six weeks' fruitless trial, were closed. The grievous want of success which attended these liberal, well-intentioned, and well-devised, but tardy measures for the benefit of the suffering poor deserves more than a passing notice. The causes of it are, I fear, not of local or of recent origin, nor confined to Calcutta, but are prevalent all over Bengal. Enslaved by the trammels of a degrading religion, by which their thoughts are chained, their reasoning faculties hood-winked, and their natural affections thwarted, all offers of even the most simple, obvious, and unquestionable temporal advantage to the recipient, and of undeniable and acknowledged liberality and goodwill on the part of the bestower, if involving the slightest deviation from ancient usage, are received by the Bengallee, not with the modest and thankful accord of silent gratitude, but with timid and suspicious, more than Trojan horror, perchance with the apathetic declaration of entire submission to the decrees of fate. If accepted at all, it is with the unblushing demand for pecuniary reward, for compliance with 'master's orders,' or 'master's pleasure,' or with the fawning and false semblance of a pleased acquiescence."

The number of admissions only amounted to 69, being the lowest outcasts of society, of whom 32 died. Happily, however, the epidemic soon afterwards declined.

The population of Calcutta is of an extraordinary kind, inasmuch as it is *stationary*. A careful census in 1837 stated the entire population of all creeds, castes and countries at 229,714, while a somewhat *less* number was found by a census of 1843. A stranger is struck by the paucity of children and old men found in the streets and at the doors; but this, as well as the stationary character of the population, is explained by the fact, that the majority of Hindoo labourers, estimated by Dr. Stewart at 175,000, dwell six or seven miles from the town, crossing or crowding the river Hooghly on their way to Calcutta in the morning and returning thence at night. Even of the dwellers in the town the greater numbers have left their families in the country, while they resort to the capital temporarily in search of profitable occupation, as the Irish do with us. The registered births amounted in 1838 to but 2,781. During the 12 years 1832-43 the mortality was 11,045 per annum, or 5·11 per cent.—the mortality in infancy and above 60 being less in Calcutta than in England; but that between 6 and 60 being enormously greater. The total mortality of natives from small-pox in the 12 years amounted to 5625, giving an annual average of 468, the lowest year (1836) yielding but 16 deaths, the highest (1833) 2548.

*Effects of Season.*—After quoting some meteorological tables, Dr. Stewart observes that, in the course of the three Calcutta epidemics, a remarkable similarity as to the effect of season prevailed. Thus the disease progressed in the course of the winter and spring, and became checked as the rainy season commenced, entirely disappearing when this became established. The rainy season is equally adverse to the success of vaccination, and to the only other exanthemata of the country, measles and varicella. Pertussis is the epidemic of the rainy season, Remittent Fever of the autumn, while Cholera is always prevalent at change of season, especially at the setting-in of the cold weather. Dr. Stewart observes that the peculiar condition of the atmosphere which determines the type and malignancy of the epidemic of small-pox, exerts a considerable effect upon that of the epidemic which succeeds or replaces it. As the small-pox declined in May 1844, a malignant and fatal form of cholera set in, and the same has been observed in other epidemics.

*Native Treatment.*—This is cooling, starving, and expectant, more reliance being placed upon propitiating the presiding Goddess of Small-pox, *Sietula*, by incantations and offerings, than upon mundane operations. Ptisans and decoctions are given; the maturation of the pustules is encouraged by thin linseed poultices, and, when the pocks are ripe, they are punctured.

*Conclusions.*—From his own observations and those of others who have better opportunities of observing the disease among natives than he has had, Dr. Stewart concludes:—

"1. All classes and castes of natives, all ages and both sexes, are pretty equally susceptible of infection, the mortality being mainly dependent upon the modifying circumstances of previous inoculation or vaccination, of natural constitution, of present health or feebleness, of personal comfort or destitution affecting individuals; and by the particular constitution of the atmosphere, the salubrity of the locality, and the construction of the dwelling. 2. The great majority of the victims are totally unprotected either by vaccination or previous inoculation, though the latter practice is most common. 3. Those who have undergone the disease previously, either naturally or communicated by inoculation, or who have been *successfully* vaccinated, always have the disease in a somewhat *modified* form. The incursive fever is often equally violent, but the eruptive stage is always milder, and the secondary fever proves fatal only in previously debilitated or scrofulous subjects. 4. In those who suffered from high fever at the onset, with much cerebral and venous excitement, head-ache, delirium, and severe lumbar pain, unless early destroyed by fever, the eruptive stage succeeds most fully and favourably, affording, though in a confluent form, great relief to the sufferings, and promise of a favorable termination. In these cases the chief danger arises about the 12th or 14th day from the secondary fever then occasioned by the erythematous condition of the skin. 5. The worst and most certainly fatal cases are those of asthenic type occurring generally in miserable, impoverished, and debilitated subjects, or in lethargic, obese and scrofulous constitutions, in which early passive hæmorrhages, the result of venous congestion, occur from the mucous surfaces of the bladder and bowels, or from the uterus in women, in which the maturative process is imperfect, and petechiæ or bloody vibices soon cover the skin, death ensuing about the eighth day from anemia, while the sensibilities are fully retained to the last moment. The violence of the epidemic is moderated by a damp and hot atmosphere, its diffusion promoted by dry and cold weather."

The Europeans and East Indians suffer very slightly from the disease when compared with the Natives; but most of the subjects of it had been vaccinated. An interesting case of the co-existence and simultaneous progress of variola and vaccinia, and another of petechial small-pox, are related.

2. *The Disease among Cows.*—We must dwell upon this section very briefly. Great numbers of these animals suffered from a fatal disease, termed by the natives "Mattah" or inward small-pox, during the raging of the small-pox epidemic. None inspected by Dr. Stewart had any pustular eruption. He has likewise quite failed during many efforts at repeating Mr. Ceely's experiments of inoculating the cow. So, too, among many hundreds of cows inspected, he has only been able in three instances to discover anything like papulæ, vesicles, or pustules on the teats or udders of the animal—and in these could not communicate the virus to children. No medical or veterinarian professor in Calcutta had seen the cow-pox among cows in England. From the 1st. Sept. 1843, to 1st June 1844, there were reported to have died 2233 head of cattle of "Mattah;" and as the native butchers were said to kill for sale all animals they suspected of having taken the disease, the fear of eating infected meat induced the European and East Indian population to abstain from beef with all the rigour of the Hindoos themselves. The poultry also died by hundreds at this time. A report by Dr. Hosack of a choleroid disease which prevailed in New York in 1842, caused by eating smoke-dried beef, prepared from diseased animals, is referred to; and some of the meat offered for sale at a low price to the lower orders in Calcutta is described as diseased and unfit for food, while the slaughter-houses were kept in a disgraceful condition. A

great desire is expressed that Calcutta should be supplied with properly constructed and inspected *abattoirs*, as at Paris and New York. We sincerely hope that no corporation vested interests will oppose there, as they do here, this improvement.

*Vaccination in Bengal.*—When, as we have seen in a former part of this article, that even in England, and scarcely in any country of Europe, has vaccination had free scope given it to do its goodly work, we can feel no surprise that its success has been still less in India. The climate, the religious prejudices and diversified character of its inhabitants, their practice of inoculation, and their imperfect intercourse with Europeans, alike tend to this end. The vaccine virus, after numerous disappointments, succeeded at last in India; and Dr. Shoolbred in his Report, stated that 8140 subjects were vaccinated in Bengal in 1804. Still, at several of the Stations, the supply soon became lost, and its recovery was a matter of great difficulty, until Dr. Shoolbred employed the *dried scabs* as the mode of conveying it, as recommended by Mr. Bryce. These proved nearly as potent as the recent lymph itself.* Dr. Cameron's Report, published in 1831, proved that vaccination was progressing, although slowly, and denied the existence of any cases of variola supervening after its proper performance.

In 1832, Mr. M'Pherson discovered the genuine cow-pock among some cattle at Moorshebad. A virus of apparently superior power was thus obtained and freely distributed. In 1836, Messrs. Brown and Furnell believed they had discovered the genuine cow-pock in Assam, and several children were successfully vaccinated; but unfortunately from the lymph obtained from some of these a form of small-pox was communicated. The vaccine virus loses much of its power in Bengal upon the approach of the rainy season, recovering it as the cold weather returns. Dr. Stewart replaced the old 1802 virus by some newly received from England in Sept. 1839, and which proved very satisfactory in its effects. All his correspondents agree in considering it as superior to that before in use.

*Re-vaccination.*—Among the European society there have occurred not a few cases this year of modified small-pox, in persons of adult age, who had been vaccinated in England in early life, and very many persons have been re-vaccinated with complete success. Of 87 natives, however, re-vaccinated by the author, only five were successful.

*Present State of Vaccination in Bengal.*—Tables are given shewing the progress of vaccination in Bengal, from 1827 to 1843 inclusive. The total number vaccinated amounted to about 652,000, the greatest number,

---

* The utility of these crusts is repeatedly alluded to in the course of the book, and the author observes, in relation to the failure of his early employment of them—"I received a hint, however, from my friend Mr. Martin (along with two very fine crusts taken from his own child in London and inclosed in his letter), which was—to levigate the crusts finely and *dilute them largely*, using about eight or ten drops of water at least to each *after* reducing them to pulp. I found that, in this way, I succeeded perfectly in every case."

68,680, occurring in 1843. The author states his conviction that the practice is making way with the natives; although some of their inoculators are propagating the disease by inoculating for the small-pox at the rate of 600 per annum each. Still the number above-mentioned, considering the enormous population of Bengal, must be considered pitifully small. Considerable sums of money have been expended in endeavouring to propagate the blessing: and much of the author's work is taken up with criticising the present mode of rendering governmental assistance, and suggesting improvements, which, from the local character of the knowledge required to appreciate them, cannot interest our readers.

"Reviewing the whole history of Vaccination in Bengal, I fear it must be owned that its progress has been slow, that its operations have been but partially successful, and that its present state and prospects are unsatisfactory and discouraging. Neither can it be denied that public opinion has been unsettled regarding its advantages, and public confidence shaken in its efficacy and permanence as an antidote to small-pox—a feeling which is not confined to the common people, but prevails more perceptibly among the better-informed and reflecting classes of the community, and is not without participators among the profession itself."

The reports of the Superintending Surgeons in the different Divisions of the Presidency unite in stating that the numbers vaccinated are quite disproportionate to the expense incurred. The vested interests of the priesthood, to whom inoculation is a profitable monopoly, and the ignorance, and apathy of the natives, are alike unfavorable to progress. Inducing the Brahmins to substitute the practice of vaccination as a source of emolument has however been several times successfully resorted to.

According to a long anonymous account quoted with approbation from the Lancet of 1834, it would seem that vaccination had been much more systematically and successfully pursued in the *Bombay Presidency*. Native vaccinators were employed under the superintendence of well-paid travelling English superintendents. An ill-judged economy has, however, of late much diminished the salaries of the latter.

According to a Report forwarded from the *Madras Medical Board*, vaccination is also in a much more thriving condition in that than in the Bengal Presidency, above two million persons having been vaccinated in the ten years, 1831-40. This extensive system of operations was carried out by 162 native vaccinators under the supervision of 33 superintending surgeons. The natives in this part of India are said not to be prejudiced against vaccination, believing it of a similar nature to the inoculation they had always been accustomed to—the chief obstacle arising from their apathy. Dr. Stewart, in commenting upon the differences observed in the two presidencies, attributes them to—1. The greater prejudices of the Bengallee. 2. The assistance derived by the vaccinators from the Madras Revenue Officers. 3. The climate of Bengal being unfavorable for the preservation of the virus, which is only transmissible in cool weather. 4. It is only in large towns, as Calcutta, where a sufficiently large and mixed population can secure sufficient subjects for transmission, and where the force of example can discourage inoculation. 5. The returns of the native vaccinators are by no means to be implicitly relied on. Dr. S. suggests that operations in Bengal should be confined to large towns; and that the practice of vaccination should be recommended to the notice of natives by *permanently associating it with that of Medicine*, performing it publicly at hospitals and dispensaries under the personal superintendence of the surgeons, and offering a small reward for the preservation of the crusts.

To revert for a moment to our own country, we may, in conclusion, express our opinion—

1st. That, although the Vaccination Extension Act is a step in the right direction, no permanent good can result until *vaccination is rendered compulsory*. Even now the Tables of Mortality teem with accounts of children snatched away by small-pox, having been cruelly denied the benefits of protection; and in the first Quarterly Table for this year we find the following pertinent remarks in reference to Norwich and Ashton.

"Few of the victims of small-pox had been vaccinated; and thus in one city between 200 and 300 persons were suffered to perish in three months—others were maimed, blinded, and deformed for life—through negligence of the parents in the application of the protection discovered by Jenner, and placed at the disposal of all by the Legislature. Other examples of the consequences of neglecting vaccination will be found in the notes. Ashton-under-Lyne is the only parish in which it is mentioned that the churchwardens and overseers have refused to carry out the provisions of 3 & 4 Vict., to extend the practice of vaccination, by contracting with medical officers for the gratuitous protection of the poor. Sixty-one funerals took place in Ashton from small-pox. In Jenner's life, an instance is mentioned of the cost of coffins convincing the overseers of a certain parish of the advantages of vaccination, after all the higher arguments of humanity and justice had failed."

We must confess we can see no objection to rendering this practice a *compulsory* one, and do in no-wise agree with those who would allow persons ignorant or forgetful of their duties and responsibilities to risk the lives and health of their helpless offspring, and perpetuate a dangerous disease by their carelessness and obstinacy. The fear of the ravages of an occasionally occurring pestilence, such as the cholera or plague, is deemed a sufficient reason for placing considerable restrictions on private right of action, and even upon personal liberty; and yet a disease far more deadly, because more abiding, and about the contagion of which there is no dispute, is not to be effectually met by a prophylactic which all are unanimous in praising! And this, too, not from fear of wounding any prejudices opposed to the practice which can scarcely be said to exist; but from an exaggerated dread of invading the right of private judgment, which would be all very well were it not that its exercise, or rather neglect of exercise, is at the expense of those who are too young to act for themselves, and whose protector the State should be. It is quite true that universal vaccination will not exterminate small-pox; but this admission is surely not to be employed as an argument against our obtaining the largest amount of diminution of the evil it is capable of producing.

2. That Inoculation should still be peremptorily prohibited: for if even its performance were restricted to medical men, numberless *foci* of disease must be still in course of production.

3. That at the approach of puberty, say about 10 or 12 years of age, *Re-vaccination* should be practised. From 15 to 25 is the period of life when the majority of cases of small-pox after vaccination occur. During the first quarter of last year, however, no less than 10 children died in London of small-pox after vaccination, whose respective ages were 9, 8, 6 (two), 5, 4 (three), 3 years, and one six months. Dr. Gregory, commenting on these cases in his Letter to the Registrar-General, observes,

"In reasoning on these cases, it should be borne in mind, that they are the first of the kind which have been brought before the notice of medical men. Similar occurrences may have taken place, but they have attracted no attention here or abroad. No corresponding cases have been recorded in any of the Quarterly Reports transmitted by the Provincial Registrars of England and Wales. Nothing parallel to them has occurred in the experience of the Small-pox Hospital. The earliest age at which small-pox has there been observed to prove fatal is 14 years, though several instances of the disease in a milder form have occurred at earlier dates. These considerations afford ground for supposing that lymph of an imperfect quality had been used in some, at least, of these instances."

4. That measures should be taken to obtain accurate statistical tables of the various facts relating to vaccination. This would seem to be one of the natural functions of the "National Vaccine Establishment;" but we are not aware that it has ever contributed one fact in its wretchedly meagre Reports to the advancement of our knowledge of the subject. Reference is made in that of the present year to some recent document furnished to Sir James Graham, respecting the occurrence of small-pox after vaccination; but, upon enquiry, we cannot learn that any such is accessible to the profession. This should not be, especially as former statements upon this subject by the Board have not been the most accurate possible. It is pleasing to contrast with the inertness of this body the active usefulness of the Registrar-General's department, by means of which so much invaluable information has been accumulated, and arranged, in so short a period of time. To this quarter, indeed, we can alone look for furthering inquiries upon this subject as regards the dead; but we sincerely hope that the opportunity of another census will not be passed over without some attempt being made to ascertain the exact condition of the living in respect to vaccination. We would suggest, in the mean time, that the various Registrars should be instructed to inquire, in *every case* of a child's death being registered, whether it had been vaccinated or not: and that equally whether such child has died of small-pox or not. We believe the public would be somewhat startled at the amount of non-protection which would thus be disclosed. At all events, this inquiry should always be made when death has arisen from small-pox; whereas at present it sometimes is, and sometimes is not, made.

Sir Matthew Tierney's *brochure* is a *little* out of date, seeing that the latest information contained in it comes down only to 1804, and that the aspect of the question has undergone some alteration since then.

